# Laser-Induced Breakdown Spectroscopy Coupled with Multivariate Chemometrics for Variety Discrimination of Soil

**DOI:** 10.1038/srep27574

**Published:** 2016-06-09

**Authors:** Ke-Qiang Yu, Yan-Ru Zhao, Fei Liu, Yong He

**Affiliations:** 1 College of Biosystems Engineering and Food Science, Zhejiang University, 866 Yuhangtang Road, Hangzhou, 310058, China; 2 College of Mechanical and Electronic Engineering, Northwest A&F University, Yangling, 712100, China; 3 Key Laboratory of Equipment and Informatization in Environment Controlled Agriculture, Ministry of Agriculture, P. R. China

## Abstract

The aim of this work was to analyze the variety of soil by laser-induced breakdown spectroscopy (LIBS) coupled with chemometrics methods. 6 certified reference materials (CRMs) of soil samples were selected and their LIBS spectra were captured. Characteristic emission lines of main elements were identified based on the LIBS curves and corresponding contents. From the identified emission lines, LIBS spectra in 7 lines with high signal-to-noise ratio (SNR) were chosen for further analysis. Principal component analysis (PCA) was carried out using the LIBS spectra at 7 selected lines and an obvious cluster of 6 soils was observed. Soft independent modeling of class analogy (SIMCA) and least-squares support vector machine (LS-SVM) were introduced to establish discriminant models for classifying the 6 types of soils, and they offered the correct discrimination rates of 90% and 100%, respectively. Receiver operating characteristic (ROC) curve was used to evaluate the performance of models and the results demonstrated that the LS-SVM model was promising. Lastly, 8 types of soils from different places were gathered to conduct the same experiments for verifying the selected 7 emission lines and LS-SVM model. The research revealed that LIBS technology coupled with chemometrics could conduct the variety discrimination of soil.

Soil has an extremely complex chemical elemental composition and highly diverse^1^,^2^, as it contains many constituents like minerals, organic matters, living organisms, fossils, air, and water. It also includes many classes of organic compounds spanning a large molecular weight range and including carbohydrates, aromatics, starches, nitrogen-containing compounds, and fatty acids^1^. The physical, biological, and chemical properties of soils could change significantly as a result of human activities such as habitation and farming. Considering the diversity of soil contents, quality and usability, a systematic scientific study on the soil’s elemental composition and type is of great concern^2^,^3^. Detection of abundance or deficiency of soil elements and identification of soil types are the key points of information acquirement tools in precision agriculture^4^, and it also provides a theoretical basis for prevention of soil polluted by heavy metal and sustainable development of agriculture.

Conventionally, discrimination of soil types was mainly depended on observation of geomorphic characteristics (color, grain size, appearance and other physical properties) of soil, which would be finished by professional staff and effected by human’s subjectivity^5^. Researchers demonstrated that soil fertility status and crop productivity could be considered as a basis for crop management and soil variation^6^. Chemical analysis, including atomic absorption spectrometry (AAS), X-ray fluorescence spectroscopy (XRFS), inductively coupled plasma-atomic emission spectrometry (ICP-AES), of elemental composition and other substances was an effective method for classifying different soil, which was high-cost and time-consuming.

Laser-induced breakdown spectroscopy (LIBS), also named laser induced plasma spectroscopy (LIPS) or laser ablation spectroscopy (LAS), a kind of atomic emission spectroscopy, has been considered to be a future “superstar” for green chemical analysis due to its unique features^2^,^7^,^8^,^9^,^10^,^11^, like fast analysis time, multi-element detection in any kind of material (solid, liquid, and gas), high spatial resolution (at the μm range), and the potential to carry out *in-situ* or stand-off analysis^9^,^12^,^13^. Relying on the unique capability, LIBS technique has witnessed tremendous growth and been widely applied in variety of fields, such as environmental monitoring^14^,^15^, archaeological investigations^16^, geological applications^17^, biomedical detection^18^,^19^, industrial analysis^20^, agriculture^21^,^22^,^23^, food^24^,^25^,^26^, and space exploration^27^,^28^.

At present, the calibration curve method with single band data mining and calibration free method were widely used for concentration measurement in LIBS analysis^29^. Recently, multivariate chemometrics methods combined with LIBS technology showed a sharp growth in the field of soil analysis. Examples of soil analyzing based on multivariate chemometrics mainly included predication of element concentrations in soil and classification of soil variety. For determining the elemental content, multivariate analysis methods, such as partial least-squares regression (PLSR), artificial neural network (ANN), support vector machine (SVM), random forest regression (RFR), multi-linear regression (MLR), principal component regression (PCR), standard addition method (SAM), were widely applied to LIBS data^30^,^31^,^32^,^33^,^34^,^35^,^36^. On the other hand, researchers developed a variety of ways to deal with the variety discrimination of material. In general, identification of emission lines of a significant element or use of multiple LIBS emission lines (calculating intensity ratios of the selected lines) were the two methods for classifying the soil samples^29^. Moreover, multivariate analyses have been already widely applied to LIBS data for classification purposes^37^,^38^, such as classification of slag sample using partial least squares discriminant analysis (PLS-DA)^33^, discrimination of sedimentary rocks based SVM^39^, principal component analysis (PCA) and PLS-DA for distinguishing characteristics of the geological samples^40^, PCA on data of polluted soils using a mobile LIBS system^41^, rock identification using three methods of PCA, PLS-DA and soft independent modeling of class analogy (SIMCA)^42^, PCA for plastic classification^43^, adjusting spectral weightings (ASW) for polymer identification^44^, rock classification by remote LIBS using independent component analysis (ICA)^45^, hierarchical cluster analysis (HCA) for classifying the chicken tissue (brain, lung, spleen, liver, kidney and skeletal muscle)^46^, classification of pharmaceutical tablets based on SIMCA^47^, classification of toys relying on toxic elements using the k-nearest neighbors (KNN)^48^. However, few studies on variety classification of soil using LIBS coupled with multivariate analyses have been reported.

In the current study, LIBS technique was employed to carry out the variety discrimination of soil. Multivariate chemometrics method of PCA, SIMCA and LS-SVM were introduced to conduct the characteristic and discrimination analysis, and then the selected characteristic spectral lines and optimal discriminant model were verified.

## Results and Discussion

### Overview of soil LIBS spectra

The obtained LIBS spectra curves contained more than 17,000 wavelength channels in a wavelength range starting at 300 nm in the ultraviolet (UV) and extending into the near-infrared (NIR) to 850 nm.

**Figure 1:**
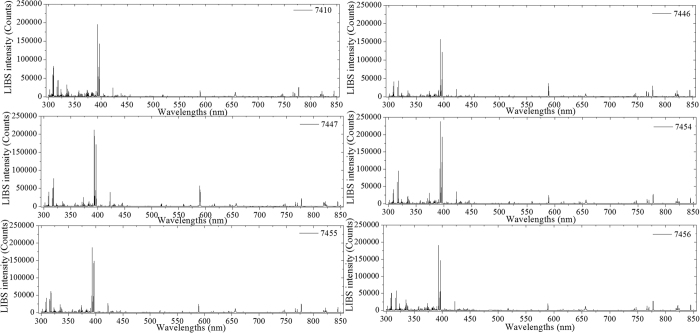
Representative LIBS spectra curves of six types of soil samples in 300–850 nm.

Figure 1 shows the original LIBS spectra of the 6 types of soil samples (GBW07410, GBW0746, GBW07447, GBW07454, GBW07455, and GBW07456) in 300–850 nm. It could be observed from Fig. 1 that there were similar profiles of curves of 6 soils. However, the discrepancy of 6 groups only appeared on the different LIBS spectra intensity. In detail, most of the valuable and high-intensity spectral lines were around the region of 300–450 nm. Concurrently, LIBS spectra in 450–850 nm exhibited a relatively low-intensity and stable tendency, except several obvious peaks around the wavelengths at 590 nm, 655 nm, 770 nm, and 820 nm.

**Figure 2:**
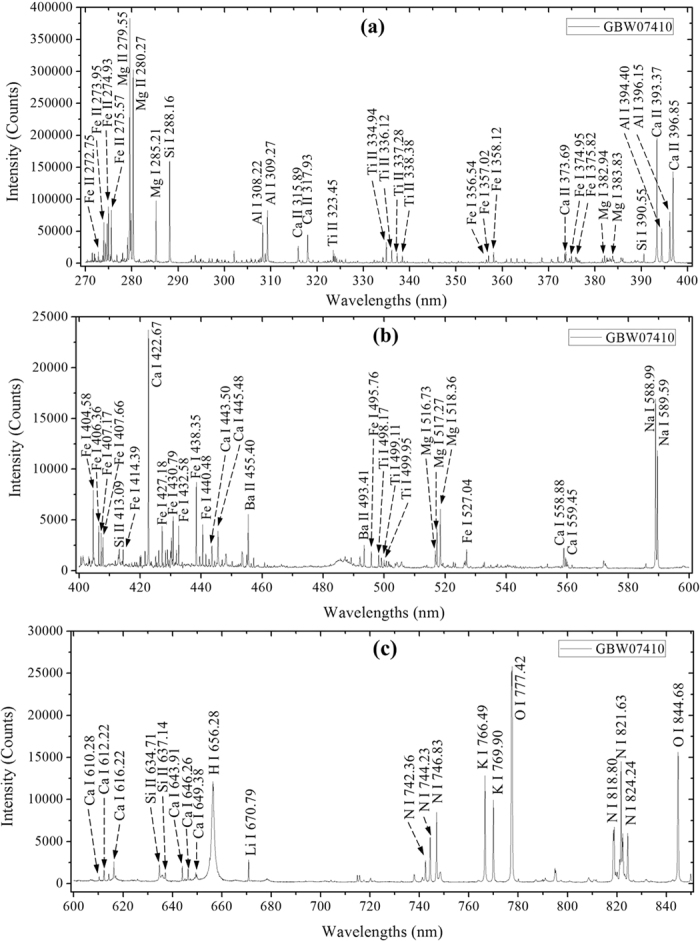
The ownership of main emission lines in LIBS spectrum of soil sample numbered GBW07447 in (**a**) 300–400 nm, (**b**) 400–600 nm, and (**c**) 600–850 nm (I: atomic spectral lines and II: ionic spectral lines).

From Fig. 1, 6 types of soil samples with similar LIBS spectra curves were attributed to their homologous matrix of chemical and elemental composition^11^,^49^. In order to analyze information of the LIBS spectral lines, the soil sample numbered GBW07410 was taken as an example to identify those spectral lines according to NIST Atomic spectra database and Kurucz database^50^. Figure 2 illustrated a typical spectrum of soil samples numbered GBW07410. Most of emission lines standing for different elements were accurately identified and labeled in corresponding positions.

In Fig. 2, a number of emission lines (atomic and ionic spectral lines) with different LIBS intensity were observed to contain the information of Al, Ca, Si, Fe, Mg, Na, Mn, Li, Ti, N, K, Ba, H, and O element. The spectral lines of O and N might be caused by O_2_ and N_2_ in the air and the by chemical substances in soil sample.

The identified emission lines of elements Al, Fe, Mg, Ca, Na, K, and Si in the oxide components present in the soil sample are listed in Table 1. These emission lines had minimal interference from other emission lines and provided enough LIBS intensity.

### Principal components analysis (PCA) on LIBS data

Because the soil samples had different matrix and experiment conditions (especially the Echelle spectrometer was sensitive to temperature) changed, which might bring the discrepancy of LIBS data. Hence, it was necessary to conduct the LIBS data preprocessing. Normalization had a widely application in preprocessing of the LIBS data. Area normalization is a kind of normalization; its purpose is to “scale” samples in order to get all data on approximately the same scale. So, in order to compensate for spectral changes caused by matrix effects and variation in experimental conditions, all spectra were normalized by using area normalization method, accomplishing an equal area under the curve for each spectrum^51^,^52^.

In this study, the acquired LIBS spectra included 17,173 wavelength channels in 300–850 nm. Classification using all the spectra could enhance the calculation time and increase the requirements of equipment performance for LIBS measurements. Hence, removal of highly superfluous variables and selection of few crucial emission lines of elements were of significance for multivariate analysis.

**Figure 3:**
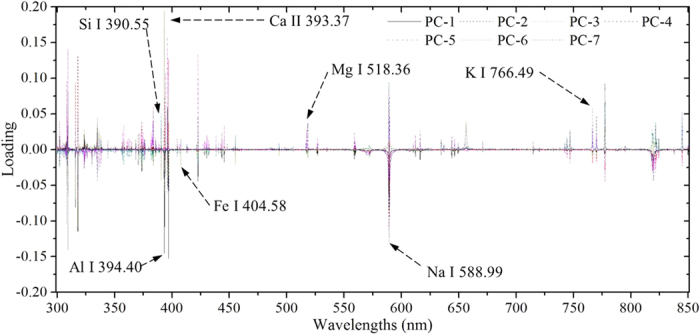
The loading plot of first seven PCs from PCA on full spectra of six soil samples.

First, PCA was employed to transform the full spectra into several principal components (PCs) and the loading plot was executed to select the important emission lines. The first seven PCs explained 96.19% of the variations of original all spectral information and their loading plot are shown in Fig. 3. It could be observed that most emission lines of main elements (Al, Fe, Mg, Ca, Na, K, and Si) offered the relatively large loading coefficients, which were in alignment with the labeled lines in Fig. 3.

Finally, the emission lines with high signal-to-noise ratio (SNR) would be selected to conduct the further analysis. Based on above, a total of 7 characteristic lines (marked in Fig. 3) at Si I 390.55 nm, Al I 394.40 nm, Fe I 404.58 nm, Mg I 518.36 nm, Na I 588.99 nm, Ca II 393.36 nm, and K I 766.49 nm, which contained LIBS spectral peaks of Si, Al, Fe, Mg, Na, Ca, and K of each soil class, were chosen from the identified emission lines. Then, a matrix with 180 × 7 (LIBS spectra × lines) was obtained to implement the next analysis.

**Figure 4:**
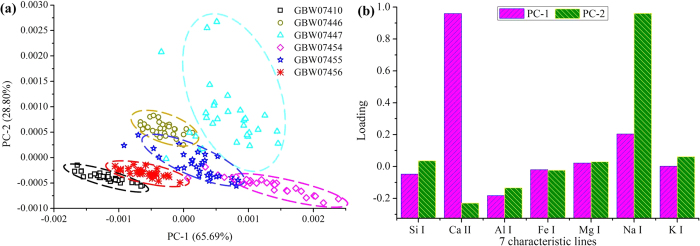
The score (**a**) and loading (**b**) plots of first two PCs from PCA on LIBS spectra at the selected emission lines of six soil samples.

Next, another PCA was carried out using the LIBS spectra at the selected characteristic lines to display any variation among the 6 types of soil sample. The first 2 PCs explained 94.49% (PC-1: 65.69% and PC-2: 28.80%) of the variations among total spectral information, and their score and loading plots are shown in Fig. 4. Each point in the scatter plot (score plot) represented one spectrum. Fig. 4(a) showed that an apparent clustering could produce with PC-1 and PC-2. The LIBS spectra of soil were distinguished in the side of PC-1, while some spectra tended to be on the positive side of PC-2. Meanwhile, there was a slight cross between the classes of GBW07446, GBW07447, GBW07454, and GBW07455. It was worth noticing that there was an obvious difference between 6 groups of soil samples.

Figure 4(b) showed the loading plot of the PCA, which also revealed the importance of the analyzed variables. It could be concluded that Ca and Na elements gave expression to dominating contribution on PC-1 and PC-2, respectively. For fully explaining the scatter of score plot, loading plot of Fig. 4(b) and Table 4 were combined to analyze the scatter distribution of 6 types of soils. The GBW07454 and GBW07447 classes with relatively high concentration of Ca element were located in the positive side of PC-1; and the GBW07410 class with relatively low content of Ca was situated in the negative side of PC-1. In addition, the classes of GBW07446 and GBW07447 containing relatively high concentration of Na element distributed in the positive side of PC-2; and classes (GBW07410, GBW07456, GBW07455, and GBW07454) with similar content of Na were scattered in negative side of PC-2. The classes of GBW07446, GBW07447, and GBW07455 exhibited some slightly intersections, which was attributed to their approximate content of Al and K.

Although some differences could be observed in Figs 1 and 4, chemometrics methods were employed to extract and concentrate the connotative information for further discriminating soils^53^.

### Soft independent modeling of class analogy (SIMCA)

For SIMCA analysis, SPXY (sample set partitioning based on joint x-y distances) method proposed by Galvao *et al*.^54^, was first implemented to divide the data matrix (180 × 7) of LIBS spectra and corresponding labeled classes of each spectrum into a calibration set with 120 LIBS spectra and a predication set with 60 LIBS spectra. SIMCA was applied to calibration set of the LIBS spectra. As mentioned principle of SIMCA, a separate PCA was performed for every class of soil, resulting in 6 individual PCAs. For the predication of the “unknown” LIBS spectra, these PCA models were applied with 4 PCs each, except for the model of class GBW07454, where 3 PCs were used. Then, a SIMCA model was established using the LIBS spectra of the calibration set. Putting the data of predication set into the SIMCA model could compute the forecast results.

**Figure 5:**
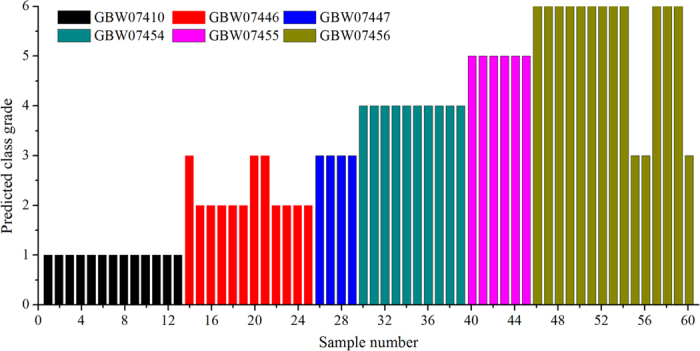
Bar plot of SIMCA model for the predication set of 60 predicated LIBS spectra. Predicted class IDs were as follows: 1, GBW07410; 2, GBW0746; 3, GBW07447; 4, GBW07454; 5, GBW07455; and 6, GBW07456.

The result of SIMCA classification and predication of unknown class samples is shown in Fig. 5. Nearly all the LIBS spectra of soil in the predication set were correctly classified, except 6 ones were misclassified. 3 LIBS spectra of GBW07446 and GBW07456 were identified as the class of GBW07447, respectively. This resulted in an overall correct classification accuracy of 90.00% (54 vs. 60), which indicated that those selected emission lines (Si I 390.55 nm, Al I 394.40 nm, Fe I 404.58 nm, Mg I 518.36 nm, Na I 588.99 nm, Ca II 393.36 nm, and K I 766.49 nm) had a reliable discrimination power for distinguishing 6 groups of soils.

### Least-squares support vector machine (LS-SVM)

Next, LS-SVM methodology was employed to establish models based on the calibration set (same to SIMCA model) for discriminating the 6 classes of soils. To obtain an excellent classification performance, two factors of regularization parameter γ and the RBF kernel function parameter σ^2^ in LS-SVM classifier have to be optimized. The parameter γ could determine the tradeoff between maximizing the model performance and minimizing model complexity, and the σ^2^ was the bandwidth and implicitly defined the nonlinear mapping from input space to some high-dimensional feature space^55^,^56^,^57^. Based on LS-SVM model, its classified results of predication set (60 spectra) are summarized in a confusion matrix presented in Table 2. The numbers of correctly classified samples were listed on the diagonal, and the off-diagonal was the misclassification. Meanwhile, the sum of the numbers in each column was the number of samples examined for each type. From Table 2, all the analytical spectra of soil samples in 6 classes were correctly discriminated to their own groups, resulting in a correct classification rate of 100%. The results suggested that LIBS technology had a potential to discriminate different types of soil combined with proper chemometrics method, demonstrating the capability of chemometrics-LIBS in field of spectroanalysis.

### Evaluation of discrimination models

The results of 2 models could be concluded that LS-SVM method provided more accurate discrimination results compared with the SIMCA model. Then, receiver operating characteristics (ROC) curve was employed to evaluate the performance of 2 models. From the ROC curves of SIMCA and LS-SVM discriminant models, the parameters of “area” and “std” in SIMCA ROC curve were 0.93695 and 0.008533, while those factors were 1 and 0 in LS-SVM ROC curve. The results indicated that the discrimination capability of LS-SVM was superior to SIMCA model. This might arise from the fact that LS-SVM as a nonlinear method has an ability to overcome the variability in LIBS measurements and showed better performance in handling high-dimensional LIBS data sets compared to conventional linear method^38^, like SIMCA in this study.

### Verification of the selected emission lines and discrimination model

In order to verify the reliability of the selected emission lines and the accuracy of discrimination model, 8 representative types of soils were collected from different places in China. Those soils included: cinnamon soil in Luoyang city of Henan province (HN-LY), moisture soil in Taian city of Shandong province (SD-TA), lime concretion black soil in Zhumadian city of Henan province (HN-ZMD), terra rossa in Changde city of Hunan province (HN-CD), paddy soil in Hanzhong city of Shaanxi province (SX-HZ), cinnamon soil in Jinzhong city of Shanxi province (SX-JZ), red soil in Hangzhou city of Zhejiang province (ZJ-HZ), yellow soil in Fuyang city of Zhejiang province (ZJ-FY). After removing impurities, all the soil samples were taken to the lab and air-dried (or oven-dried at 60 °C). Then, a series of processes of grinding, sieving (100 mesh), weighing, about 3 g soil power was pressured into pellet using a presser as mentioned in section of Soil samples in Materials and methods. Based on the same parameters of LIBS device and data acquisition mode (detailed in section of experimental device and LIBS data acquisition in Materials and Methods), a total of 440 LIBS spectra (8 soil classes, 55 analytical spectra per classes) were recorded in the database. Then, the obtained LIBS spectra at 7 characteristic lines (Si I 390.55 nm, Al I 394.40 nm, Fe I 404.58 nm, Mg I 518.36 nm, Na I 588.99 nm, Ca II 393.36 nm, and K I 766.49 nm) were selected, forming a matrix of 440 × 7 (LIBS spectra × lines) to be used for the further analysis.

**Figure 6:**
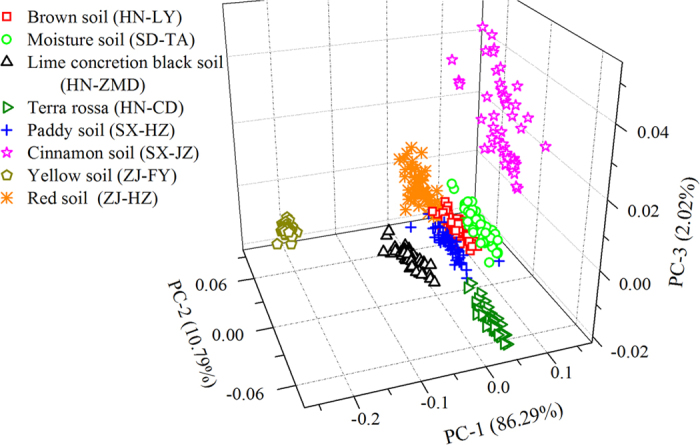
The score plot of first three PCs from PCA on LIBS data of eight types of soil samples in different places.

To explore the discrepancy of 8 soils, PCA was adopted on the obtained matrix of LIBS spectra. PCA had compressed most variance of spectra into the first 3 PCs, which explained 99.10% variance of original data. Figure 6 shows the score plot of the first 3 PCs. It could be seen that most points of LIBS spectra of each type were clustered together and the boundaries of different types were relatively clear. So, it could be concluded that there was an obvious differentiation in 8 types of soil and the selected 7 characteristic lines were valid to distinguish the different soils.

LS-SVM model was also developed for discriminating the 8 types of soils. The acquired spectral matrix was split into a calibration set (290 spectra × 7 emission lines) and a predication set (150 spectra × 7 emission lines) by the SPXY method with the ratio of 2:1. The sample labels in the database, which were integer varying from 1 to 8, were considered as the class labels used for producing models. Using the spectra in the calibration set, the classification performances approached 100% based on the LS-SVM. To assess the performance of this model, ROC curve of the LS-SVM classifiers displayed the parameters “area” and “std” of 1 and 0, respectively. It could be seen that the LS-SVM model obtained excellent discriminant performances. The above reliable discrimination results indicated that it was feasible to discriminate the LIBS spectra of different types of soils by means of LS-SVM methodology.

## Conclusions

This research focused on investigating the characterization of soil utilizing LIBS technology combined with chemometrics methods. Based on the features of soil’s LIBS curves and PCA on full spectra, several characteristic lines were identified. In order to simplify the discriminant model, 7 emission lines (Si I 390.55 nm, Al I 394.40 nm, Fe I 404.58 nm, Mg I 518.36 nm, Na I 588.99 nm, Ca II 393.36 nm, and K I 766.49 nm) with high SNR were selected to conduct the further next analysis. PCA was carried out on the LIBS spectra at the 7 selected emission lines. An obvious cluster was observed and analyzed. Then, SIMCA and LS-SVM discrimination models were established, and their performances were evaluated by ROC curve. Results demonstrated that the LS-SVM model was the optimal model for discriminating the different types of soils. Moreover, the 7 selected emission lines and the LS-SVM model were applied to the other 8 types of soil samples, which also achieved outstanding discrimination results. To improve the extendibility of our application, more samples and a diversified analytical data set should be taken into account to obtain enough spectrum data in further investigations. It could provide a theoretical guidance for establishing agrotype system and farmland management.

## Materials and Methods

### Experimental device and LIBS data acquisition

**Figure 7:**
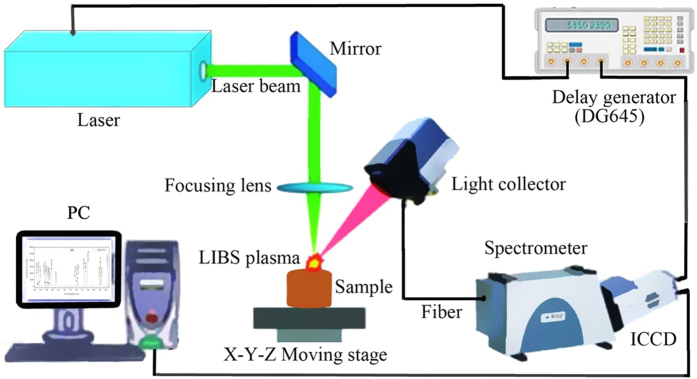
A representative LIBS analytical system setup for soil analysis.

A typical LIBS system illustrated in Fig. 7 was assembled in our lab using the following main components: a Q-switched Nd: YAG laser (Vilte-200, Beamtech Optronics Co. Ltd., Beijing), a high resolution Echelle spectrometer (Mechelle 5000, Andor Technology) coupled to an intensifier charge coupled device (ICCD) camera (iStar DH340T-18F-03, Andor Technology), a delay generator (DG645, Stanford Research Systems, USA), an X-Y-Z moving stage (Zolix Instruments Co. Ltd., Beijing), and a personal computer (PC) with the Andor SOLIS software (Version 4.25, Andor Technology).

The experiments were finished in air. Before collecting LIBS data, the system was warmed up for 0.5 h to ensure the thermal stability of the instruments. Then, the setup was corrected by a Hg: Ar lamp (Ocean Optical, HG-1, Hg-Ar lines 253–922 nm) for wavelength calibration and a Deuterium-Halogen light source (DH-2000-BAL, Germany) for intensity calibration.

After that, the Q-switched Nd:YAG laser operating with 1 Hz repetition frequency and 7 ns pulse duration emitted a laser pulse with energy of 80 mJ at wavelength of 532 nm. Through the reflection of a mirror, the laser beam was focused vertically onto the soil sample surface with a 100 mm focal distance lens. Then, LIBS emission was collected by the light collector and delivered by optical fiber to the Echelle spectrometer (200–975 nm, 195 mm focal lengths, F/7, resolution of λ/∆λ 6000) equipped with a time-gated ICCD camera (1024 × 1024 pixels, 13.6 × 13.6 μm^2^/pixel). The delay generator provided a proper delay time to eliminate the initial continuum emission. In order to obtain fresh locations to be ablated, the soil pellets were placed on the sample holder which could be moved automatically in *X, Y* and *Z* directions by stepper motors. For all measurements, the gate width and exposure time of the ICCD were set to 2 μs and 0.01 s, respectively. A gate delay of 2.5 μs, which was the gap between the laser pulse and the start of gating time, was chosen. In addition, microchannel plate (MCP) gain was fixed at 500.

For each spectrum, an accumulation of 20 laser pulses per site was collected to increase the signal-to-noise ratio (SNR). To minimize the influence of sample heterogeneity and laser energy fluctuations^52^, 180 shots were performed from 9 sites (9 spectra were obtained) for each sample. Then, the spectra of each 3 sites were averaged into an analytical spectrum, and 3 representative LIBS spectra could be acquired from one soil sample. Meanwhile, 30 LIBS spectra were recorded from each class (10 samples per class). According to the preceding process, a total of 180 LIBS spectra were gathered from 6 classes of soil samples.

### Soil samples

In this research, the certified reference material (CRM) of soil powder sample (grain size is less than 0.075 mm) was provided by National Institute of Metrology, P. R. China. The selected soil samples included 6 classes: black soil (GBW07410) from Heilongjiang province, sandy soil (GBW07446) and saline-alkali soil (GBW0747) from Inner Mongolia Autonomous Region, loess (GBW07454) from Shaanxi province, sediment (GBW07455) from the Huaihe River in Anhui province, and the other sediment (GBW07456) from the Changjiang River in Jiangsu province. The certified elemental compositions of the main oxide material (SiO_2_, Al_2_O_3_, Fe_2_O_3_, FeO, MgO, CaO, Na_2_O, K_2_O) in CRM of 6 soils are presented in Table 3 (other elemental compositions are not listed). The certified elemental compositions were measured by the Institute of Geophysical and Geochemical Exploration of the Chinese Academy of Geological Science using X-ray fluorescence (XRF), inductively coupled plasma-mass spectrometry (ICP-MS), atomic absorption spectroscopy (AAS), etc.

Meanwhile, the concentrations of main elements (Si, Al, Fe, Mg, Ca, Na, K) in oxide were calculated and listed in Table 4.

To obtain the homogeneous surface of the soil for laser ablating, the soil powder was made into cylindrical pellets using a manual pellet presser (FY-24, SCJS Co., LTD, Tianjin, China). In detail, the applied load of the presser was 15 Mpa lasting for 4 minutes. Each soil pellet had a weight of 3 g, a diameter of 25 mm, and a thickness of 3 mm.

### Multivariate chemometrics methods

PCA is an unsupervised technique (classes or composition of the samples in the data matrix is not involved) and has a wide application in reducing the dimension of multivariate data sets^39^,^58^. The principle and application of PCA could be found in the paper reported by the literature of^1^,^59^,^60^. Meanwhile, the score plot of PCs is used to reveal the features of variable distribution^61^, and the loading plot of PCs can exhibit the importance of different variables.

Least-squares support vector machine (LS-SVM), an optimized version of the standard SVM, is a powerful methodology in pattern recognition and function estimation^55^,^56^,^62^,^63^,^64^. In this research, radial basis function (RBF) kernel function was adopted to establish the LS-SVM model, it was convenient to detect the effect of independent variable (*X*) on dependent variable (*Y*) based on analysis of linear regression coefficient. The details of LS-SVM could be found in the literatures of^56^,^62^,^63^.

Soft independent modeling of class analogy (SIMCA), an application of PCA, is a supervised technique for classification^30^,^48^,^59^,^65^. In this model, the acquired data set is subdivided corresponding to class affiliation, and then a separate PCA model is performed independently for each of those classes. Meanwhile, numbers of PCs are selected individually for the corresponding classes. Samples with unknown class membership are then assigned to a class by projecting them into each subspace. Then, the residual variances of unknown samples are compared to judge which categories the sample belongs to^66^,^67^.

Lastly, confusion matrix with an advantage to obtain true values and false values from the result^68^ could be used to list the discrimination results. Meanwhile, receiver operating characteristics (ROC) curve, a useful tool for organizing classifiers and visualizing their performance^69^,^70^,^71^, is employed to assess the performance of discrimination models.

### Software tools

The processes of statistical calculations and data analyses were carried out by “The Unscrambler X10.1” (CAMO PROCESS AS, Oslo, Norway) and MATLAB 7.8 (R2009a) software (The MathWorks, Inc., Natick, MA, USA). In addition, Origin Pro 8.0 SR0 (Origin Lab Corporation, Northampton, MA, USA) software was used to design graphs. All processes were run on a PC (CPU: Intel Core i3-3220 @3.30GHz, RAM: 4.00GB) under Windows 7.

## Additional Information

**How to cite this article**: Yu, K.-Q. *et al*. Laser-Induced Breakdown Spectroscopy Coupled with Multivariate Chemometrics for Variety Discrimination of Soil. *Sci. Rep.* **6**, 27574; doi: 10.1038/srep27574 (2016).

## Figures and Tables

**Table 1:** The emission lines and corresponding spectral regions of the main elements in soil.

**Elements**	**Emission lines** (**nm**)
Al	I 308.21, I 309.27, II 394.40, II 396.15
Fe	I 356.54, I 357.02, I 358.12, I 404.58, 406.36, I 407.17, I 427.18, I 430.79, I 432.58, I 438.35, I 440.48
Mg	I 382.94, I 383.83, I 517.27, I 518.36
Ca	II 315.89, II 317.93, II 373,69, II 393.37, II 396.85, I 422.67, I 443.50, I 445.48, I 588.88, I 610.28, I 612.22, I 616.22, I 643.91, I 646.26, I 649.38
Na	I 588.99, I 589.59
K	I 766.49, I 769.90
Si	I 390.55, I 413.09

**Table 2:** Results of LS-SVM model for classifying LIBS spectra of soil samples in predication set.

**Number**	**GBW07410**	**GBW07446**	**GBW07447**	**GBW07454**	**GBW07455**	**GBW07456**
GBW07410	13	0	0	0	0	0
GBW07446	0	12	0	0	0	0
GBW07447	0	0	4	0	0	0
GBW07454	0	0	0	10	0	0
GBW07455	0	0	0	0	6	0
GBW07456	0	0	0	0	0	15

Rows: actual classification of samples, and columns: classification by LS-SVM model.

**Table 3:** The certified elemental compositions of the oxide component in CRM of six soils (in wt. %).

**Number**	**SiO**_**2**_	**Al**_**2**_**O**_**3**_	**Fe**_**2**_**O**_**3**_	**FeO**	**MgO**	**CaO**	**Na**_**2**_**O**	**K**_**2**_**O**
GBW07410	65.64 ± 0.38	14.55 ± 0.18	4.60 ± 0.13	–	1.25 ± 0.04	1.42 ± 0.10	1.90 ± 0.09	2.59 ± 0.07
GBW07446	78.30 ± 0.33	9.65 ± 0.09	2.07 ± 0.03	0.50	0.78 ± 0.08	1.83 ± 0.05	2.31 ± 0.04	2.56 ± 0.03
GBW07447	60.40 ± 0.26	10.56 ± 0.05	3.63 ± 0.05	1.10	2.58 ± 0.07	6.80 ± 0.10	3.05 ± 0.09	2.11 ± 0.02
GBW07454	60.93 ± 0.25	11.76 ± 0.13	4.30 ± 0.07	1.30	1.99 ± 0.05	7.18 ± 0.10	1.74 ± 0.03	2.28 ± 0.02
GBW07455	66.15 ± 0.4	11.73 ± 0.19	4.00 ± 0.08	1.20 ± 0.16	1.87 ± 0.06	4.59 ± 0.07	1.90 ± 0.03	2.18 ± 0.04
GBW07456	58.87 ± 0.65	13.15 ± 0.16	6.12 ± 0.09	1.70	2.75 ± 0.08	4.91 ± 0.07	1.22 ± 0.03	2.37 ± 0.04

**Table 4:** The concentrations (mg·g^−1^) of main elements in oxide of six soils.

**Number**	**Si**	**Al**	**Fe**	**Mg**	**Ca**	**Na**	**K**
GBW07410	306.80 ± 1.77	77.01 ± 0.95	32.17 ± 0.89	7.54 ± 0.24	10.15 ± 0.71	14.10 ± 0.67	21.50 ± 0.58
GBW07446	365.98 ± 1.55	51.08 ± 0.48	18.37 ± 0.21	4.70 ± 0.48	13.08 ± 0.36	17.14 ± 0.30	21.25 ± 0.25
GBW07447	282.31 ± 1.22	55.89 ± 0.26	33.94 ± 0.20	15.56 ± 0.42	48.60 ± 0.71	22.63 ± 0.67	17.52 ± 0.17
GBW07454	284.79 ± 1.17	62.25 ± 0.69	40.17 ± 0.28	12.00 ± 0.30	51.32 ± 0.71	12.91 ± 0.24	18.93 ± 0.33
GBW07455	309.19 ± 1.86	62.09 ± 0.99	37.30 ± 1.82	11.28 ± 0.36	32.80 ± 0.50	14.10 ± 0.23	18.10 ± 0.33
GBW07456	275.16 ± 3.04	69.60 ± 0.85	56.01 ± 0.63	16.58 ± 0.48	35.09 ± 0.50	9.05 ± 0.22	19.67 ± 0.33
